# Role of a Ketogenic Diet on Body Composition, Physical Health, Psychosocial Well-Being and Sports Performance in Athletes: A Scoping Review

**DOI:** 10.3390/sports8100131

**Published:** 2020-09-23

**Authors:** Amy-Lee Bowler, Remco Polman

**Affiliations:** 1 Faculty of Health Sciences and Medicine, Nutrition & Dietetics, Bond University, Gold Coast QLD 4226, Australia; 2 Faculty of Health, School of Exercise & Nutrition Sciences, Queensland University of Technology, Brisbane QLD 4059, Australia; remco.polman@qut.edu.au

**Keywords:** ketogenic, athletes, body composition, physical health, well-being

## Abstract

Background: Recently, a focus has been placed on investigating the potential benefits of adherence to a ketogenic diet in enhancing body composition, physical health, psychological well-being, and performance of athletes from various sporting disciplines. As the available research is yet to be collated and analyzed in a single review, this scoping review aims to analyze and draw conclusions from the available literature that exists on the efficacy of a ketogenic diet among athletic populations. Methods: Several primary research databases and any relevant citation lists were searched to locate appropriate studies for inclusion in this scoping review. Studies that investigated the effects of adherence to a ketogenic diet (KD), defined by a carbohydrate intake of less than 5% of total energy intake, on body composition, physical health, psychological well-being, and performance among an athletic population were included in the review. From 814 articles screened, 12 were identified as meeting the inclusion criteria and were included in the final scoping review. Results: Adherence to a KD has beneficial effects on body weight and fat mass. Varying effects were identified on physical health with the diet, eliciting positive effects on fat oxidation but potentially deleterious effects on stool microbiota and iron metabolism. Conflicting results were reported regarding the effects of a KD on sporting performance. Benefits were reported regarding athlete well-being following commencement of a KD, but only after week two. Conclusions: The results of this scoping review demonstrate that there are both beneficial and detrimental effects associated with adherence to a KD among athletic populations. It is understood that further research is required to make any concrete recommendations regarding a KD to athletes.

## 1. Introduction

Over the past 60 years, an emphasis has been placed on the consumption of adequate amounts of carbohydrate pre-exercise/training to ensure adequate muscle glycogen content. It has long been understood that sufficient muscle glycogen stores are required for optimal sporting performance. Advice from major sports nutrition organizations has reflected this ideology, promoting carbohydrate-focused diets for athletes [1].

More recently, an interest in low-carbohydrate alternatives, including the ketogenic diet (KD) has increased. Similar to a low-carbohydrate, high-fat diet, the KD induces an augmentation in free fatty acids circulating within the body. The diet elicits this physiological response by involving a restriction in dietary carbohydrate (<5% of energy intake per day), elevation of fat intake (>60% of dietary intake), and adequate protein consumption. This restriction in dietary carbohydrate is associated with relative decreases in glucose availability, inducing a physiological metabolic state known as ketosis [2]. Ketosis is characterized by an increase in circulating ketone bodies, namely, acetoacetate (AcAc) and B-hydroxybutyrate (BHB). BHB is acknowledged as the chief ketone body within the peripheral tissues and is the transporter of energy to these tissues [2]. Thus, the elevation of blood BHB levels above 0.5 mmol/L [2] within the body are utilized to demonstrate the presence of nutritional ketosis. During nutritionally induced ketosis, these particular ketone bodies are used as an alternative to glucose and become the main energy source for the brain and other bodily tissues [2].

The KD has gained significant attention due to its potential as a medical intervention for epilepsy [3,4] and its ability to induce weight loss and body composition changes in obese and overweight individuals [5,6,7]. In more recent times, a KD has generated interest among the sports nutrition community, with evidence suggesting that a reduction in dietary carbohydrate and increases in circulating ketone bodies results in a shift in substrate utilization during submaximal exercise bouts, namely, from glycogen to body fat [8].

A preliminary search of the academic literature did not indicate that a systematic review has taken place to examine whether a KD in athletes’ influences body composition, physical health, psychological well-being, or sports performance. As such, we conducted a scoping review to summarize the current literature to provide an overview of the effects of adherence to a KD. This review sought to determine the effects that occur within athletic populations on both short- and long-term body composition, physical health, psychological well-being, and sports performance in athletes.

## 2. Materials and Methods

### 2.1. Methods

This scoping review has been conducted in accordance with the Joanna Briggs Institute (JBI) methodology for scoping reviews [9,10].

### 2.2. Search Strategy

For the purposes of this scoping review, we accessed a number of primary health research databases (PubMed, Embase, CINAHL, Web of Science, Scopus, SPORTDiscus, and Cochranes Database) in conjunction with a review of citation indexes and reference lists to locate peer-reviewed studies relevant to the research question.

The search was conducted from inception until June 2020 and considered only English manuscripts. The search terms used to explore the databases were ketogenic, keto, very low carbohydrate, athlete, sport, and exercise.

### 2.3. Inclusion Criteria and Outcome Measures

In order to be deemed appropriate for inclusion in this scoping review, studies must have investigated the effects of a KD, defined by a protocol that involves dietary contribution from carbohydrate of less than 5% of energy intake per day, or blood ketone levels of >0.5 mmol/L BHB [2], and have been implemented longer than or equal to 21 days. Studies were considered if they included male or female participants who were either trained or well-trained in their chosen sport. Reported outcomes may encompass a range of areas including, but not limited to, body composition (i.e., fat mass and lean body mass), physical health (i.e., blood composition, pH, iron status, immune function), psychological well-being (i.e., perceived wellness), and sporting performance (e.g., laboratory- and field-based test performance). In addition, the length of study, outcome measure, and the instrument used to measure the outcomes was reported.

For this review, it is important to clarify how health and well-being were defined when searching the current literature. As this scoping review focused on athletic populations, we decided that various measures of health and well-being specific to athletes would be examined. Body composition, namely, body fat and body weight, were investigated, as this is a particularly important measure of health in athletes that is conducive to their overall performance. Other measures of physical health such as metabolic characteristics (i.e., fat oxidation, circulating metabolites, and muscle glycogen availability), blood profile (i.e., lactate levels, bicarbonate concentrations, iron levels, and blood pH), heart rate, and exercise performance (i.e., maximal exercise capacity and strength) were all considered when searching for appropriate studies. Psychological well-being was identified as pertaining to athletes’ perceptions of the ketogenic diet and their overall thoughts and feelings regarding this dietary intervention and the potential effects on their psychological well-being, training, and exercise performance.

### 2.4. Data Extraction

After applying the inclusion criteria to the studies obtained from each of the relevant databases, we obtained data on study citation details (author, title, journal, and publication year), context of the study (sport involved), participants (age, gender, and sample size), dietary protocol (length and macronutrient distribution), and final outcomes, all of which was entered into an Excel spreadsheet.

Each article was grouped by the outcome measured (body composition, physical health, sports performance, athlete well-being), with three articles measuring more than one outcome. For these articles, each outcome measure was represented separately in the appropriate group.

Due to the nature of the research question, the literature included in the final qualitative evaluation predominantly consisted of studies that employed either a crossover design, non-randomized allocation to a dietary intervention group, or pre–post testing methods. Following selection, each of the articles were critically appraised against the relevant JBI or Centre for Disease-Based Management Critical Appraisal Checklist and were deemed appropriate for inclusion in the final scoping review.

## 3. Results

### 3.1. Search

**Figure 1:**
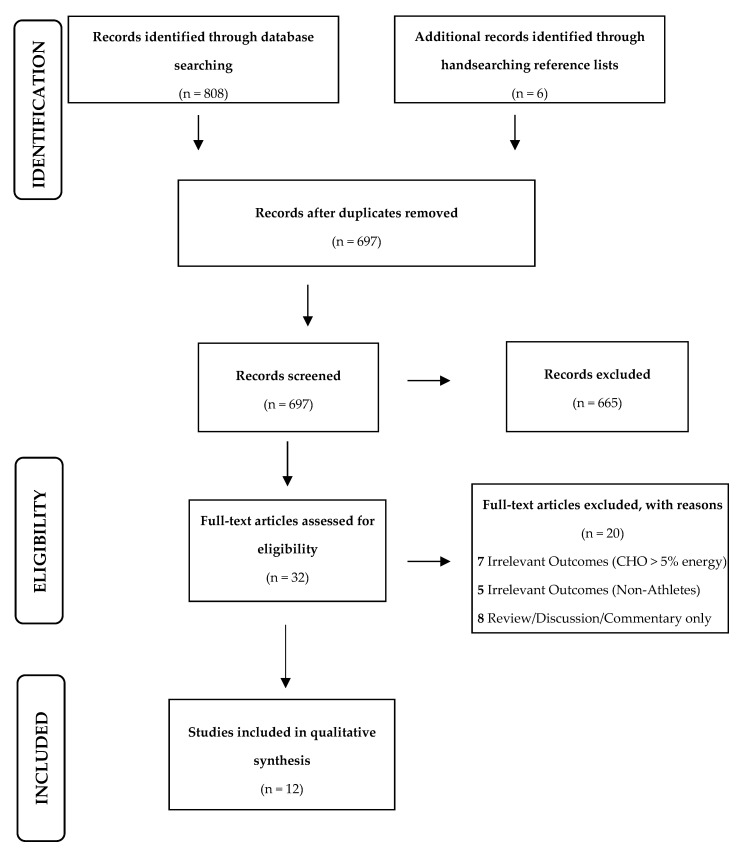
Prisma flowchart describing the study selection pathway.

The search revealed a total of 814 articles related to the search terms, however, only 12 articles met each of the criteria stipulated for article inclusion (see Figure 1). After removing 117 duplicate articles, a further 665 articles were also deemed unsuitable for inclusion, as these involved non-athletic populations, included carbohydrate intakes >5% total energy intake, or involved dietary protocols that were <21 days in duration. The topics that were included in the final review were body composition, physical health, sports performance, and athlete well-being.

### 3.2. Ketogenic Diet

The characteristics of each of the articles included in this scoping review are displayed in Table 1. The total sample was comprised of 101 athletes (84 males, 17 females) from seven sports. The athletes ranged in age from 15 to 41 years.

As detailed in Table 1, each of the relevant studies involved dietary protocols that restricted carbohydrate to less than 5% of energy intake per day. In 10 of the included studies, fat intake was above 75% of total dietary intake. Additionally, in two of the studies, protein intakes were considerably high (40% of total daily energy intake). Of the 12 studies included in the final review, seven investigated the effects of a KD over a three-week period, four ran over a four-week period, and one considered the effects of a KD over a 12-week period.

### 3.3. Effect of a KD on Various Health and Well-Being Outcomes

**Figure 2:**
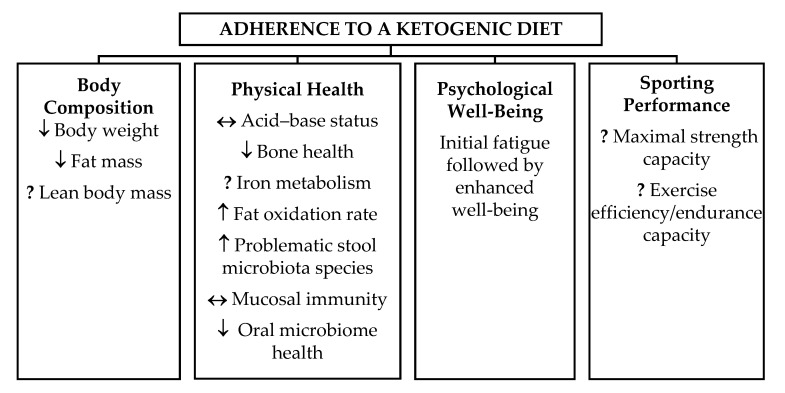
Summary of the effects of a ketogenic diet (KD) on various health and well-being outcomes.

After reviewing each of the articles selected for inclusion in this review, it was evident that equivocal results existed regarding adherence to a KD. Figure 2 provides a brief summary of the effects of a KD on various health and well-being outcomes. A KD was seen to exhibit desirable effects on body weight and fat mass in athletes [11,12], however, the effect on lean body mass is yet to be confirmed. No adverse health outcomes were observed on the acid-base status [13] or mucosal immunity [14] of athletes but increases in potentially problematic stool microbiota species [15] were identified. Adherence to a KD tended to have detrimental effects on endurance performance [11,16] but further research is required to determine the true effect of a KD on actual sporting performance.

## 4. Discussion

The aim of this scoping review was to examine the effects of adherence to a KD on body composition, physical health, psychosocial well-being, and sports performance in athletes. The review indicates that, to date, only a small number of studies have investigated the effect of a KD on the body composition, physical health, psychological well-being, and sports performance of various athletic populations, which has been heterogenic in nature.

### 4.1. Ketogenic Diet and Body Composition

Two of the studies obtained for review examined the effects of adherence to a KD on body composition using either bio-electrical impedance analysis (BIA) [11] or skinfold assessment [12]. Similar to studies in obese and overweight individuals’, researchers found improved body composition indexed by reductions in body weight (PRE 69.6 ± 7.3 kg; POST 68.0 ± 7.5 kg) and fat mass (PRE 5.3 ± 1.3 kg; POST 3.4 ± 0.8 kg) among a group of elite artistic gymnasts who adhered to a KD [12]. Lean body mass and muscle mass remained relatively unchanged following the KD protocol among the gymnasts. In a group of taekwondo athletes [11], researchers also observed significant reductions in body weight (PRE 64.11 ± 7.19 kg; POST 60.34 ± 6.59 kg) and body fat percentage (PRE 12.59 ± 3.59; POST 12.21 ± 3.59) following adherence to a KD, but these results were similar in the non-KD group. Although these results provide promising findings for manipulation of body composition within athletic populations, they should be interpreted with caution. While BIA has shown promise for the estimation of body composition in athletes, it may underestimate body fat percentage and overestimate fat-free mass [17,18]. Similarly, skinfold assessment merely predicts body composition via estimation equations and assumptions and is influenced by accurate identification of sites and use of calipers [19,20].

While the current literature on athletic populations is inconclusive, it is important to acknowledge that several studies conducted on non-athletic individuals have demonstrated that Lean body mass (LBM) tends to be retained following a KD [21,22,23]. A study on 10 healthy men demonstrated that adherence to a KD over 6 weeks resulted in retention of LBM at week three followed by an increase at week six [23]. This may be due to the energy (29 kcal/kg/day) and protein intakes (2 g/kg/day) that were maintained by participants throughout the duration of this study [23]. On the basis of these findings, it is suggested that future studies on athletic populations concentrate on protein intake or other supplementation (e.g., creatine or leucine) to ensure adequacy for LBM retention and/or increases when commencing a KD.

### 4.2. Ketogenic Diet and Physical Health

#### 4.2.1. Acid-Base Status

Acid-base status can have deleterious effects on the body’s buffering mechanisms, resulting in metabolic acidosis. This can cause adverse health effects including kidney stones and be detrimental to athletic performance. In a non-randomized observational study conducted on elite-level race walkers, the researchers found no significant differences in blood pH, bicarbonate concentrations, or lactate levels following adherence to a 21-day KD [13]. This suggests that adherence to a KD does not have any detrimental effects on acid-base status nor does it increase the risk of metabolic acidosis among athletic populations.

#### 4.2.2. Bone Health

Optimal bone health is imperative to athletes that are training and competing in high-level sports. Often, athletes competing at elite levels are susceptible to bone injury, partly due to dietary factors such as low energy availability and/or the strenuous nature of the exercise program. A recent study conducted by Heikura et al. [24] investigated the effects of a KD on bone health among a group of elite-level race walkers. After a 3-week intervention period, this study found that athletes had increased markers of bone resorption (+22% CI 9, 35%) and decreased markers of bone formation (−14% CI −19, −9), when compared to baseline values. These results were also observed post-exercise. This suggests that adherence to a KD may have deleterious effects on bone modeling and bone re-modeling markers, both at rest and during high-intensity exercise bouts.

#### 4.2.3. Iron Metabolism

When exercise is performed in the presence of limited glycogen stores, certain inflammatory markers such as interleukin-6 (IL-6) are elevated [25]. IL-6 regulates the release of hepcidin, which in turn maintains healthy physiological levels of iron stores. Inflammation increases hepcidin levels, potentially disrupting iron absorption. Each of these responses are likely exhibited post-exercise. Given the associations between limited glycogen stores, IL-6 elevation, hepcidin, and iron stores, it is possible that dietary interventions involving decreased carbohydrate intake may exacerbate the disruptions that occur to iron metabolism post-exercise [25].

A study with competitive race-walking athletes [25] conducted over a 3-week period showed significantly higher post-exercise IL-6 (KD PRE 0.6 ± 0.8 pg/mL, POST 10.8 ± 0.8 pg/mL; CHO PRE 0.7 ± 6.6 pg/mL, POST 6.3 ± 0.6 pg/mL) among the KD intervention group compared with those in the carbohydrate-rich dietary group, potentially due to limited muscle glycogen stores. While these findings may suggest that athletes who adhere to a KD may be at risk of iron deficiency, it has been acknowledged that these results are not uncommon following periods of strenuous or specific training regimes. Nonetheless, as iron is a key functional constituent of hemoglobin and myoglobin, it is required for oxygen uptake, transport, and energy production [26]. Iron deficiency may prove detrimental to athletes as it may result in reduced oxygen uptake, transport, and energy production, thus having the potential to impair physical health and performance [27]. However, further research is required to fully understand the associations between a KD and iron status.

#### 4.2.4. Fat Oxidation and Metabolism

In a study on CrossFit-trained athletes [28], adherence to a KD displayed varying results regarding fat oxidation rates between male and female athletes. Among males, a KD resulted in an increase in the rate of fat oxidation, however, this was only significant at lower exercise intensities (i.e., 35% VO_2 max_ habitual diet ≈ 0.006 g/min/kg FFM, KD ≈ 0.0095 g/min/kg FFM; 50–65% VO_2 max_). In females, the rate of fat oxidation was significantly higher at 80% VO_2 max_ (habitual diet ≈ 0.002 g/min/kg FFM, KD ≈ 0.009 g/min/kg FFM).

Studies conducted on both elite race walkers and runners have demonstrated that adherence to a KD results in increases in fat oxidation rates. Among endurance runners [16], maximal fat oxidation and the relative intensity at which maximal fat oxidation occurs both significantly increased following a KD (PRE 0.57 ± 0.10 g/min vs. POST 1.12 ± 0.10 g/min and PRE 43% ± 5% VO_2_ max vs. POST 70% ± 4% VO_2_ max). Similar changes in fat oxidation rates were exhibited among a group of elite race walkers, with peak maximal fat oxidation rates of 1.57 ± 0.32 g/min attained during 2 h of walking at 80% VO_2_ peak [29].

An increased fat oxidation rate is reflective of increased lipolysis of adipose tissue and increased storage of fatty acids in skeletal muscle tissue in the ketogenic condition. Interestingly, it appears that the differences in fat oxidation rates between genders may be attributed to the higher levels of circulating estrogen among female athletes [30]. It is understood that estrogen affects physiological mechanisms responsible for fatty acid oxidation, however, further research in this area is required to fully understand the mechanisms by which fat oxidation rates differ between genders.

#### 4.2.5. Stool Microbiota

Athletes exhibit different gut microbiota profiles to non-athletic individuals [31]. This may be attributed to differences in dietary intakes, including increased protein intake that is common among athletic populations. Murtaza et al. [15] reported that a KD elicited significant decreases in relative concentrations of *Faecalibacterium* spp. and *Bifidobacterium* spp., both of which have been reported as being beneficial for maintaining intestinal homeostasis [32]. *Faecalibacterium* spp. is also known to stimulate anti-inflammatory effects when in abundance. As the diversity of the gut microbiome is associated with several physiological processes that affect the health and well-being of an individual, it is understood that reductions in *Faecalibacterium* spp. and *Bifidobacterium* spp. could affect the physical health of an athlete [32,33].

The same study [15] found that consumption of a KD was positively correlated with increases in levels of *Dorea* spp. and *Enterobacteriaceae* in elite race walkers. It has been reported that Enterobacteriaceae is often elevated with the prevalence of various disease states within the human body, while raised levels of *Dorea* spp. have been positively associated with total cholesterol and low-density lipoprotein (LDL) concentrations albeit in hyperlipidaemic rats [34].

The findings outlined in this study indicate that elevated selective pressure on the gut microbiota is observed among individuals who adhere to a KD when compared with those who consume high or periodized carbohydrate diets [15]. Studies have highlighted the potential consequences of alteration to the gut microbiome such as the development of digestive pathologies, metabolic diseases, neurological disorders, cancers, immune conditions, and skin problems [32]. Additionally, this particular study also demonstrated the negative associations between abundances of *Dorea* spp. and exercise economy. This suggests there are potentially detrimental effects associated with changes in gut microbiota such as decreased athletic performance due to reduced exercise economy and, possibly, reduced overall physical health among elite race walkers. It is important to note, however, that research on the gut microbiome is still emerging and evidence pertaining to the negative effects of a KD on athlete microbiota remains in the preliminary stages.

#### 4.2.6. Mucosal Immunity

Salivary immunoglobulin-A (s-IgA) is known to be a marker of the immune system, with decreases in S-IgA reflective of a reduction in immune function and increased risk of upper respiratory illness. In a study on 26 elite race walkers [14], researchers observed that regardless of dietary protocol, s-IgA concentration increased post-exercise. There were no significant differences between diet groups for resting s-IgA concentration, flow rate, or secretion rate. In all groups, there was a moderate to large increase in s-IgA concentration between pre-and post-testing periods (KD 49 ± 45%, high-carbohydrate 90 ± 77% and periodized-carbohydrate 77 ± 24%). Moderate increases were also evident in all diet groups for resting secretion rate, but this finding was equivocal. As this particular study involved a period of intensified training, it is understood that the results obtained indicate that increased training load may negate any potential effects that carbohydrate manipulation may have on the mucosal immunity of athletes [14]. In saying this, due to the equivocal results of this study, results should be interpreted with a degree of caution.

#### 4.2.7. Oral Microbiome

Among the same group of participants within which stool microbiota was examined, the researchers also analyzed the oral microbiome to determine the effects of a KD [35]. In humans, the oral cavity has been identified as a possible contributor to the onset and progression of certain chronic ailments such as diabetes, cancer, and cardiovascular disease [36]. Currently, studies have also highlighted the potential for changes in the oral microbiome to elicit disruptions to nitric oxide homeostasis, thus possibly resulting in increases in blood pressure [37]. The study conducted by Murtaza and associates [35] showed that KD decreased amounts of *Haemophilus*, *Neisseria*, and *Prevotella* spp. and increased the abundance of *Streptococcus* spp. Alterations to these particular species of microbiota are thought to reduce the performance benefits often experienced from supplementation of nitrate/beetroot juice. These potentially detrimental effects are thought to be attributed to the impairment of the nitrate–nitrite NO axis that occurs following a KD [38].

### 4.3. Ketogenic Diet and Psychological Well-Being

Only one study in endurance athletes [14] examined how adherence to a KD influenced well-being. This study reported some initial decreases in perceptions of general health and physical readiness and lower mood. However, this only lasted for a few weeks. Following this, athletes reported enhanced well-being, indicating that general health and physical readiness had increased significantly since baseline. There was no clear change in perceived soreness or fatigue from baseline to the end of the trial among participants in the KD group [14]. This suggests that initial adherence to a KD may affect psychological well-being negatively and that it may take several weeks for each athlete to adapt to the diet. After this time, participants report levels of psychological well-being exceeding those prior to the intervention.

### 4.4. Ketogenic Diet and Sporting Performance

The results of a KD and sporting performance appear to be equivocal and influenced by sporting type. In elite gymnasts, strength test results were maintained between baseline and at the end of the ketogenic dietary intervention [12]. This suggests that maximal strength capacity may not be influenced by a KD, however, further research is required in this area.

A study involving eight male endurance athletes demonstrated a detrimental effect on exercise efficiency, particularly at exercise intensities >70% of VO_2 max_ [16]. This decrease in exercise efficiency was indexed by increases in energy expenditure and oxygen uptake that were unable to be explained by shifts in respiratory exchange ratio. In contrast, exercise efficiency was maintained at <60% VO_2 max_ within the KD group. Time-to-exhaustion was similar for the KD group at pre- and post-testing (PRE 239 ± 27 min; POST 219 ± 53 min). Similarly, a study on five cyclists reported no significant difference in time-to-exhaustion (PRE 147 ± 13 min; POST 151 ± 25 min) between baseline testing and 4-weeks post-KD [39]. These results may indicate that submaximal exercise capacity is maintained among athletes who adhere to a KD.

Contrastingly, a study that reported on the effects of a KD on taekwondo athletes [11] found that 2000 m sprint time decreased significantly among athletes in the KD group (PRE 516.0 ± 37.7 min; POST 484.0 ± 35.6 min). The study also reported significant reductions in anaerobic fatigue assessed via the Wingate test (PRE 55.37 ± 6.16; POST 52.31 ± 7.26).

The study on elite race-walkers [30] observed a significant increase in VO_2_ peak (PRE 66.3 mL/kg/min 90% CI: 63.9, 68.7; POST 71.1 mL/kg/min 90% CI: 69.3, 72.8) following adherence to a KD, whilst at the same time, a decrease in exercise economy during a graded economy test was observed. The latter was indexed by an increase in the O_2_ demand among the KD intervention group (stage 4 VO_2_ PRE ≈ 60 mL/kg/min; POST ≈ 65 mL/kg/min), suggesting increased metabolic cost and perceived effort. Any reductions in overall exercise efficiency are believed to be attributable to an augmented O_2_ cost that results from producing energy from fat rather than carbohydrate [40].

While our review provides a brief insight into the effect of a KD on endurance and strength performance in athletes, more comprehensive reviews on this topic are available such as those published by McSwiney et al. [41], Shaw et al. [42], and Burke [43]. These reviews should be consulted for further evidence surrounding sports performance and a KD.

## 5. Limitations

Each of the studies included in this scoping review had small sample sizes (between 5 and 37 participants), reducing statistical power. Additionally, the studies that were deemed suitable for this review were of relatively low quality, employing either cross-sectional or non-randomized methodologies. Within the sports nutrition realm, it is recognized that higher quality, larger impact studies with larger cohorts are relatively difficult to perform in athletic populations as these individuals are often apprehensive to explore new dietary interventions without knowing the potential effects of these on their physical health and performance. In saying this, it is acknowledged that further studies with higher quality research methodologies are required prior to making specific recommendations surrounding the consumption of a KD among athletic populations.

Several studies included in this review were based on the same group of participants. As a few studies reported on various outcomes associated with consumption of a KD among a group of elite-race walkers, the generalizability of the results included within this review are likely limited. This also falsely places weighting on some of the negative outcomes from each of these studies, thus creating bias. This renders it particularly difficult for conclusions to be made about the efficacy of a KD across a range of different sports.

Another limitation of the studies included in this review is that they each consider the athletes’ performance in a laboratory-based environment rather than assessing actual sporting performance. Therefore, any results reported on the potential effects of a KD on sporting performance may not be truly reflective of the actual effects that may be exhibited in competition.

## 6. Practical Implications and Future Research

On the basis of these findings, athletes who find it difficult to make or maintain weight for weight-based sports may find a KD beneficial. Monitoring maintenance of LBM or implementing strategies to prevent reductions in LBM (e.g., additional protein or supplementation) when starting a KD would be recommended.

The results discussed in this review aim to assist athletes in making an informed decision when evaluating possible dietary means of improving their overall body composition, physical health, psychological well-being, and sporting performance. In the future, it is recommended that researchers look at other methods of conducting studies on the efficacy of a KD such as single-subject designs or cohort studies to further validate findings.

It is important to mention that any future studies that look to explore the effects of a KD on athlete well-being also consider methods of preventing any participant dropouts that may occur over the initial stages of the dietary intervention period. As each of the studies mentioned in this review reported initial decreases in perceived well-being, future studies need to ensure that methods are employed to retain participants past this initial stage. It is recommended that future studies are conducted over longer periods of time to ascertain the exact effect a KD has on long-term health and psychological well-being. Additionally, it has been suggested throughout this review that future studies should concentrate on enhancing protein intakes to assess whether this may have beneficial effects on lean body mass and maximal strength capacity. Potentially, supplementing with leucine in future studies may prove advantageous, as studies have discussed the efficacy of leucine in stimulating muscle hypertrophy and, subsequently, strength gains in athletes [44].

Regardless of any results that have been discussed throughout this review, it is imperative that each athlete be consulted individually prior to any dietary intervention, ensuring they are provided with adequate education on the particular dietary regime to be administered.

## 7. Conclusions

This scoping review aimed to identify and synthesize the current literature surrounding the effects of a KD on the body composition, physical health, psychological well-being, and sporting performance of athletes from various sporting disciplines. This review found a small number of relevant papers, which indicates an increasing interest in the efficacy of a KD on these measures in athletic populations, but there is further research required to provide any conclusive statements regarding the efficacy of the diet. Currently, evidence suggests a positive association between adherence to a KD and body composition (reduction in body weight and fat mass) and psychological well-being (positive in the long term). However, there is equivocal evidence surrounding its effects on physical health and sporting performance.

## Figures and Tables

**Table 1:** General characteristics and main findings of the studies included in this scoping review.

Study Citation Details	Context	Participants	Dietary Protocol	Main Findings
**Body Composition**				
Paoli, A.; Grimaldi, K.; D’Agostino, D.; Cenci, L.; Moro, T.; Bianco, A.; et al.: Ketogenic diet does not affect strength performance in elite artistic gymnasts	Artistic Gymnastics	15–26 years, Male; *n* = 9	Athletes acted as own controls with adherence to KD followed by western diet. Thirty-day KD dietary protocol; 40% PRO, <5% CHO, 55% FAT.	Significant differences were exhibited in body weight (PRE 69.6 ± 7.3 kg vs. POST 68.0 ± 7.5 kg), % lean body mass (PRE 92.4 ± 1.4% vs. POST 95.0 ± 1.0%), fat mass (PRE 5.3 ± 1.3 g vs. POST 3.4 ± 0.8 g), and fat % (PRE 7.6 ± 1.4% vs. POST 5.0 ± 0.9%) following adherence to a KD. Lean body mass and muscle mass remained relatively unchanged following adherence to a KD. There were no significant differences in performance following KD intervention.
Rhyu, H.; Cho, S.: The effect of weight loss by ketogenic diet on the body composition, performance-related physical fitness factors and cytokines of Taekwondo athletes.	Taekwondo	15–17 years, Male; *n* = 20	Randomized design with assignment to either a non-KD or a KD. Three-week KD dietary protocol; 40% PRO, <5% CHO, 55% FAT.	Significant decreases were observed in weight, % body fat, FFM, and fat mass, however, there was no difference in effect between groups.
**Physical Health**				
Heikura, I.; Burke, L.; Hawley, J.; Ross, M.; Garvican-Lewis, L.; Sharma, A.; et al.: A short-term ketogenic diet impairs markers of bone health in response to exercise.	Elite race walking	24–31 years, 25 Male, 5 Female; *n* = 30	Non-randomized allocation to either a high CHO or KD. Three-week KD dietary protocol; 2.1 g/kg PRO, <5% CHO, 75–80% FAT.	Compared to baseline measures, bone resorption was increased (+22% CI 9, 35) and bone formation decreased (−14% CI −19, −9) following a KD. Bone metabolism also decreased (−25% CI −35, −14) among KD participants. Congruent results were also observed post-exercise.
Krystztof, D.; Nowaczyk, P.; Siedzik, K.: Effect of a four-week ketogenic diet on exercise metabolism in CrossFit-trained athletes	CrossFit	25–33 years, 11 Male, 11 Female; *n* = 22	Four-week ketogenic dietary protocol; 1.7 g/kg PRO, ≤5% CHO, ≥75% FAT.	Gender differences were apparent regarding fat oxidation during a KD. Males exhibited increases in the rate of fat oxidation at lower exercise intensities, i.e., 35% (habitual diet ≈ 0.006 g/min/kg FFM vs. KD ≈ 0.0095 g/min/kg FFM) and 50–65% VO_2 max_, and females experienced increases in fat oxidation rates at higher exercise intensities (85% VO_2 max_ (habitual diet ≈ 0.002 g/min/kg FFM vs. KD ≈ 0.009 g/min/kg FFM)). Males exhibited higher AUC of fat utilization and lower CHO utilization at ≤65% VO_2 max._
McKay, A.; Peeling, P.; Pyne, D.; Welvaert, M.; Tee, N.; Leckey, J.; et al.: Chronic adherence to a ketogenic diet modifies iron metabolism in elite athletes.	Elite race walking	22–32 years, 31 Male, 6 Female; *n* = 37	Non-randomized allocation (chosen by athletes) to either a high CHO, periodized CHO, or KD. Three-week KD dietary protocol; 17% PRO, <5% CHO, 78% FAT.	Total dietary iron intake was significantly lower in the KD group (13.7 ± 2.1 mg/day) than the high CHO/periodized CHO groups (17.8 ± 4.5 mg/day). Significant differences in serum ferritin from baseline to postintervention were exhibited between groups, however, there was a greater decrease among CHO diet participants (KD −23% vs. CHO diets −37%). A greater post-exercise IL-6 response was exhibited among the KD group (KD 0.6 ± 0.8 to 10.8 ± 0.8 pg/mL vs. CHO diets 0.7 ± 6.6 to 6.3 ± 0.6 pg/mL) during postintervention testing, potentially due to a decrease in muscle glycogen stores.
McKay, A.; Pyne, D.; Peeling, P.; Sharma, A.; Ross, M.; Burke, L.: The impact of chronic carbohydrate manipulation on mucosal immunity in elite endurance athletes.	Elite race walking	21–32 years, 19 Male, 7 Female; *n* = 26	Non-randomized allocation (chosen by athletes) to either a high CHO, periodized CHO, or KD. Three-week KD dietary protocol; 17% PRO, <5% CHO, 78% FAT.	Regardless of the particular dietary protocol, s-IgA concentration increased post-exercise. No significant differences observed between diet groups for resting s-IgA concentration, flow rate, or secretion rate. In regard to athlete well-being, those in the KD group reported initial decreases in perceptions of general health and physical readiness from baseline to week 2, however, this was followed by increases from week 2 to 3. There was no clear change in perceived soreness or fatigue from baseline to end of trial among participants in the KD group.
Murtaza, N.; Burke, L.; Vlahovich, N.; Charlesson, B.; O’Neill, H.; Ross, M.; et al.: The effects of dietary pattern during intensified training on stool microbiota of elite race walkers.	Elite race walking	21–32 years, Male; *n* = 21	Non-randomized allocation (chosen by athletes) to either a high CHO, periodized CHO, or KD. Three-week KD dietary protocol; 17% PRO, 3.5% CHO (0.50 g/kg/day), 78% FAT.	Adherence to a KD over a 3-week period had a greater effect on gut microbiota than adherence to a high CHO or periodized CHO diet when compared with baseline measures. Significant decreases in prevalence of *Faecalibacterium* spp. and increases in abundance of *Dorea* spp., and *Bacteroides* spp. were exhibited among the KD group, thus indicating that adherence to a KD has a significant selective pressure on athlete gut microbiota.
Murtaza, N.; Burke, L.; Vlahovich, N.; Charlesson, B.; O’Neill, H.; Ross, M.; et al.: Analysis of the effects of dietary pattern on the oral microbiome of elite endurance athletes.	Elite race walking	21–32 years, Male; *n* = 21	Non-randomized allocation (chosen by athletes) to either a high CHO, periodized CHO, or KD. Three-week KD dietary protocol; 17% PRO, 3.5% CHO (0.50 g/kg/day), 78% FAT.	Oral microbiome changed among participants in the KD group when compared with the high and periodized CHO groups. *Haemophilus*, *Neisseria*, and *Prevotella* spp. each decreased, and abundance of *Streptococcus* spp. increased following the KD intervention period.
**Sports Performance**				
Burke, L.; Ross, M.; Garvican-Lewis, L.; Welvaert, M.; Heikura, I.; Forbes, S.; et al.: Low carbohydrate, high fat diet impairs exercise economy and negates the performance benefit from intensified training in elite race walkers.	Elite race walking	21–32 years, 21 Male; *n* = 21	Non-randomized allocation (chosen by athletes) to either a high CHO, periodized CHO, or KD. Three-week KD dietary protocol; 17% PRO, 3.5% CHO, 78% FAT.	VO_2 peak_ increased significantly following 3-week adherence to a KD (66.3 90% CI: 63.9,68.7 vs. 71.1 90% CI 69.3,72.8), however, this result was negated by a significant increase in the O_2_ cost of exercise (VO_2_ ≈ 60 mL/kg/min vs. ≈ 65 mL/kg/min at graded economy test stage 4) exhibited among participants in the KD group. There was also a significant decrease in respiratory exchange ratios among the KD participants (PRE 1.10 vs. POST 0.97). Completion times for the 10 km race walk did not differ between baseline and at the end of KD protocol. Fat oxidation rates increased significantly in the KD group.
Paoli, A.; Grimaldi, K.; D’Agostino, D.; Cenci, L.; Moro, T.; Bianco, A.; et al.: Ketogenic diet does not affect strength performance in elite artistic gymnasts.	Artistic Gymnastics	15–26 years, Male; *n* = 9	Athletes acted as own controls with adherence to KD followed by western diet. Thirty-day KD dietary protocol; 40% PRO, <5% CHO, 55% FAT.	There were no significant differences in performance following KD intervention.
Phinney, S.; Bistrain, B.; Evans, W.; Gervino, E.; Blackburn, G.: The human metabolic response to chronic ketosis without caloric restriction: preservation of submaximal exercise capability with reduced carbohydrate oxidation.	Cycling	Age unknown, Male; *n* = 5	Four-week ketogenic dietary protocol; 10–15% PRO, <5% CHO, 85% FAT.	Significant differences were observed in respiratory quotient between baseline and after 4 weeks of a KD (1.04 ± 0.02 vs. 0.90 ± 0.20), thus indicating a shift in muscle substrate utilization. No significant difference in time-to-exhaustion between baseline testing and 4 weeks post-KD (147 ± 13 min vs. 151 ± 25 min, respectively).
Rhyu, H.; Cho, S.: The effect of weight loss by ketogenic diet on the body composition, performance-related physical fitness factors and cytokines of Taekwondo athletes.	Taekwondo	15–17 years, Male; *n* = 20	Randomized design with assignment to either a non-KD or a KD. Three-week ketogenic dietary protocol; 40% PRO, <5% CHO, 55% FAT.	Among the KD group, 2000 m sprint time decreased significantly (PRE 516.0 ± 37.7 min vs. POST 484.0 ± 35.6 min), with a reduction in anaerobic fatigue also reported (PRE 55.37 ± 6.16 vs. POST 52.31 ± 7.26).
Shaw, D.; Merien, D.; Braakhuis, A.; Maunder, E.; Dulson, D.: Effect of ketogenic diet on submaximal exercise capacity and efficiency on runners.	Marathon running, ultramarathon, running and triathlon	24–34 years, Male; *n* = 8	Randomized crossover design with assignment to either a habitual diet group or an isoenergetic KD. Thirty-one-day ketogenic dietary protocol; 15–20% PRO, ≤5% CHO, 75–80% FAT.	Exercise efficiency was impaired among participants in the KD group, especially at >70% VO_2 max_. This decrease in exercise efficiency was indexed by increases in energy expenditure and oxygen uptake that was unable to be explained by relative shifts in respiratory exchange ratio. Exercise efficiency was maintained at <60% VO_2 max_ within the KD group. Time-to-exhaustion was similar for the KD group at PRE and POST testing (239 ± 27 min vs. 219 ± 53 min, respectively). Fat oxidation rates increased significantly between pre- and post-KD testing.
**Athlete Well-Being**				
McKay, A.; Pyne, D.; Peeling, P.; Sharma, A.; Ross, M.; Burke, L.: The impact of chronic carbohydrate manipulation on mucosal immunity in elite endurance athletes.	Elite race walking	21–32 years, 19 Male, 7 Female; *n* = 26	Non-randomized allocation (chosen by athletes) to either a high CHO, periodized CHO, or KD. Three-week KD dietary protocol; 17% PRO, <5% CHO, 78% FAT	Those in the KD group reported initial decreases in perceptions of general health and physical readiness from baseline to week 2, however, this was followed by increases from week 2 to 3. There was no clear change in perceived soreness or fatigue from baseline to end of trial among participants in the KD group.

Note: PRO: Dietary protein intake; CHO: Dietary carbohydrate intake; FAT: Dietary fat intak; PRE: Pre-intervention; POST: Post-intervention; FFM: Fat-free mass; CI: Confidence Interval; s-IgA: Salivary immunoglobulin-A.
